# Monitoring Pre-Stressed Composites Using Optical Fibre Sensors

**DOI:** 10.3390/s16060777

**Published:** 2016-05-28

**Authors:** Sriram Krishnamurthy, Rodney A. Badcock, Venkata R. Machavaram, Gerard F. Fernando

**Affiliations:** 1 Engineering System Department, Royal Military College of Science, Shrivenham SN6 8LA, UK; sriram.k@ge.com; 2 School of Metallurgy and Materials, University of Birmingham, Birmingham B15 2TT, UK; Rod.Badcock@vuw.ac.nz (R.A.B.); rajanikanth_772001@yahoo.co.in (V.R.M.)

**Keywords:** pre-stress, composites, autoclave, extrinsic fibre Fabry-Perot, fibre Bragg gratings, Strain, temperature, residual stresses

## Abstract

Residual stresses in fibre reinforced composites can give rise to a number of undesired effects such as loss of dimensional stability and premature fracture. Hence, there is significant merit in developing processing techniques to mitigate the development of residual stresses. However, tracking and quantifying the development of these fabrication-induced stresses in real-time using conventional non-destructive techniques is not straightforward. This article reports on the design and evaluation of a technique for manufacturing pre-stressed composite panels from unidirectional E-glass/epoxy prepregs. Here, the magnitude of the applied pre-stress was monitored using an integrated load-cell. The pre-stressing rig was based on a flat-bed design which enabled autoclave-based processing. A method was developed to end-tab the laminated prepregs prior to pre-stressing. The development of process-induced residual strain was monitored *in-situ* using embedded optical fibre sensors. Surface-mounted electrical resistance strain gauges were used to measure the strain when the composite was unloaded from the pre-stressing rig at room temperature. Four pre-stress levels were applied prior to processing the laminated preforms in an autoclave. The results showed that the application of a pre-stress of 108 MPa to a unidirectional [0]_16_ E-glass/913 epoxy preform, reduced the residual strain in the composite from −600 µε (conventional processing without pre-stress) to approximately zero. A good correlation was observed between the data obtained from the surface-mounted electrical resistance strain gauge and the embedded optical fibre sensors. In addition to “neutralising” the residual stresses, superior axial orientation of the reinforcement can be obtained from pre-stressed composites. A subsequent publication will highlight the consequences of pres-stressing on fibre alignment, the tensile, flexural, compressive and fatigue performance of unidirectional E-glass composites.

## 1. Introduction

Advanced fibre reinforced organic matrix composites (AFRCs) are used extensively in applications where weight is at a premium, for example aerospace, automotive and wind energy. A number of techniques are available for manufacturing AFRCs, including filament winding, pultrusion, autoclaving, resin transfer molding (and variants), spray layup and hand layup [1]. The general production process involves the impregnation of the reinforcement or preform with a thermoplastic or thermosetting resin system. Depending on the manufacturing technique in question, the impregnated fibres or preforms are either stored until required (for example, prepregs), or used immediately (filament winding, pultrusion). In the case where semi-crystalline thermoplastic matrices are used, the polymer is heated above its glass transition temperature (amorphous polymers) or melting point (semi-crystalline polymers), shaped, consolidated and then cooled to ambient temperature. Thermoset-based preforms are generally heated to between 80 and 180 °C to initiate the cross-linking of the resin and hardener; a catalyst is used sometimes to accelerate the cross-linking reactions. These reactions result in the conversion of the liquid resin system to a highly cross-linked solid polymer that is insoluble. After a predefined processing schedule, the composite is cooled to ambient temperature.

Since the preforms associated with the production of composites are processed at elevated temperatures, residual stresses can develop in the composite due to a number of reasons [2,3,4,5,6]. Twigg *et al.* [7] proposed that the sources of residual stresses in composites can be classified as extrinsic or intrinsic. Examples of intrinsic sources of residual stresses include the following.

(i) Resin shrinkage: The crystallisation process in semi-crystalline thermoplastics is accompanied by significant shrinkage. The rate of cooling of the thermoplastic matrix and the presence of nucleating agents will influence the degree of crystallinity and hence, the magnitude of the observed shrinkage. In a thermosetting resin, the shrinkage is due to the formation of covalent bonds during the cross-linking (polymerisation) of the functional groups in the resin and hardener.
(ii) Mismatch in the coefficients of thermal expansion: Since there is generally a significant mismatch in the thermal expansions of the fibre and matrix, and adjacent plies of different fibre orientation, residual stresses will develop in the composite when it is cooled from the processing temperature.

Twigg *et al.* [8] reported that extrinsic factors such as an interaction between the preform and the processing tool, and the shape of the component can also contribute to the development of residual stresses. Other notable papers that addressed issues relating to residual stresses in organic-matrix composites can be found in references [9,10,11,12]. An overview of residual stresses in other classes of materials and the techniques for quantifying them can be found in reference [13].

The presence of residual stresses can result in the loss of dimensional stability and moreover, these stresses can be high enough to cause cracking within the matrix, even before the application of a mechanical load [14,15]. Hence, there has been significant research activity in the development of techniques to minimise or mitigate the influence of process-induced residual stresses in fibre reinforced composites. For example: (i) modification of the cure schedule [16,17,18,19,20]; (ii) electron beam curing [21]; (iii) modifying the interphase properties [22,23,24,25]; (iv) deployment of shape memory alloys [26,27]; (v) using expandable monomers [28,29] and (vi) pre-stressing the reinforcement or preform during the production of fibre reinforced composites [30]. Other processing related issues that can influence the performance of AFRCs include, fibre misalignment, waviness and voids [31,32,33].

The rationale for the current work was to develop a practical production method to reduce the magnitude of the residual fabrication strain in a unidirectional glass fibre reinforced composite. This was achieved by pre-stressing the preforms to a predetermined value prior to autoclave-based processing. The primary areas of novelty in the current work include the following:

(i) The pre-stressing rig was based on a flat-bed design thus enabling conventional vacuum-bagging and autoclave-based processing to be used. This enabled the production of void-free composites thus giving greater confidence in assessing and understanding the consequence of pre-stressing on the resultant physical and mechanical properties of the composite.
(ii) A load-cell was integrated into the rig to enable the magnitude of the applied pre-stress to be monitored and quantified.
(iii) An end-tabbing procedure was developed to ensure that only the prepregs within the end-tab regions were cross-linked or cured. This was achieved by cooling the prepregs outside the end-tab regions. This meant that the applied pre-stress was distributed uniformly over the preform. Furthermore, it enabled the relative orientation of the embedded optical fibre sensors to be maintained during processing. It is known that the displacement of optical fibres from their intended orientation can result in significant uncertainty in interpreting the output data.
(iv) Two classes of optical fibre sensors were integrated into the prepregs during the lamination stages—fibre Bragg gratings and extrinsic fibre Fabry-Perot interferometric strain sensors. Optical fibre embedment procedures and protocols were developed to ensure their survival whilst pre-stressing and subsequent vacuum bag-based autoclave processing.
(v) The embedded optical fibre sensors enabled the evolution of strain within the prepregs to be monitored in real-time during the autoclave cure cycle, and also during the subsequent cooling of the composite to ambient temperature.
(vi) Surface-mounted electrical resistance strain gauges and optical fibre strain sensors were used to quantify the magnitude of the strain released when the composite was unclamped from the pre-stressing rig.

The above-mentioned attributes represent a significant advancement over the techniques that have been reported previously for pre-stressing preforms and monitoring the evolution of residual fabrication strains during the production of fibre reinforced composites.

## 2. Review of Techniques for Manufacturing Pre-Stressed Composites

Pre-stressed composites are manufactured by applying and maintaining a pre-load on the reinforcing fibres or laminated preforms during processing. Once the processing schedule has been completed, the composite is cooled to room temperature and the previously applied pre-load is released. The following section presents a brief review of selected papers that have reported on techniques to manufacture pre-stressed composites.

### 2.1. Dead-Weight Loading

**Figure 1:**
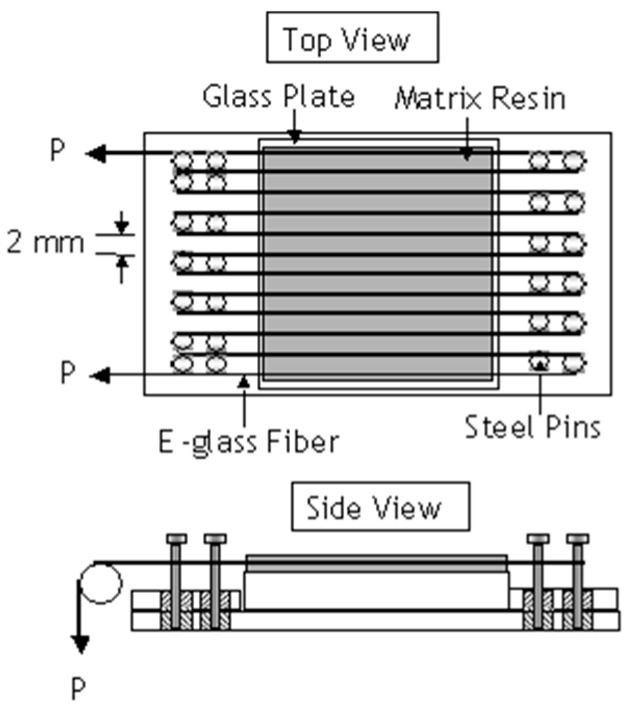
Schematic illustration of the pre-stressing method developed by Jorge *et al.* [34] where the reinforcing fibres were wound around a series of pins; the pre-load was applied using weights. “P” represents the load on the fibre bundle.

With reference to Figure 1, Jorge *et al.* [34] used a dead-weight technique to apply the required load to E-glass fibre bundles which were traversed around a series of steel pins. They prepared E-glass/polyester resin composites using a wet-layup process. The composite was cured at room temperature for twenty four hours, followed by three hours of post-curing at 80 °C. The applied pre-load was in the range 0–100 N.

### 2.2. V-Slot-Based Pre-Stressing

**Figure 2:**
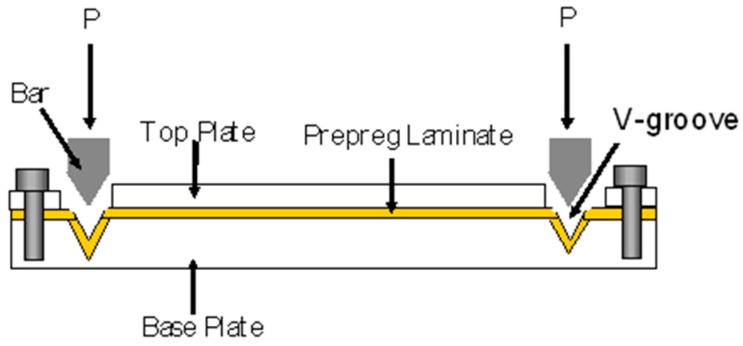
Schematic illustration of the V-slot mechanical pre-stressing method used by Schulte and Marissen [35].

Schulte and Marissen [35] used aluminium plates with two V-shaped slots and a pair of matched loading-pins to pre-stress preforms. A schematic illustration of their pre-stressing rig is shown in Figure 2. They prepared hybrid (0°-Kevlar/90°-carbon epoxy) cross-ply composites with a [0/90/90/0] lay-up sequence. The maximum pre-strain achieved using their pre-stressing rig was 1.1%. The preform was cured in a hot-press at 125 °C.

### 2.3. Filament Winding

**Figure 3:**
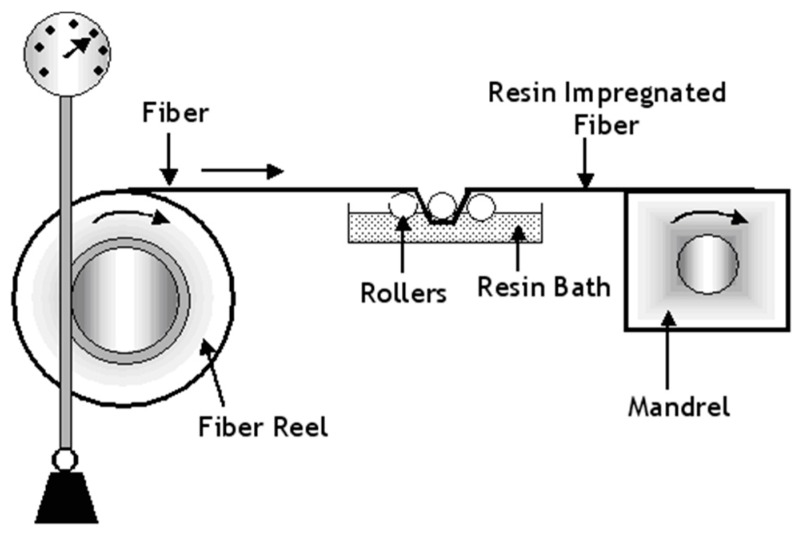
Schematic illustration of the production of pre-stressed composites using filament winding [36].

With reference to Figure 3, Hadi and Ashton [36] demonstrated that pre-stressed composites can be manufactured via filament winding. Here the tension on the fibre bundles was controlled as a means of applying the required pre-tension. In the filament winding process, resin-impregnated fibre bundles are traversed left and right continuously as they are wound around a rotating mandrel to produce the preform. The relative traverse and rotation speeds of the impregnated fibres and the mandrel respectively dictate the winding angle of the reinforcing fibres. Hadi and Ashton [36] prepared unidirectional pre-stressed composites using a flat square-sided mandrel. Rose and Whitney [37] also prepared cross-ply pre-stressed preforms using filament winding and the preforms were cured in an autoclave.

### 2.4. Hydraulic Pre-Stress Rig

**Figure 4:**
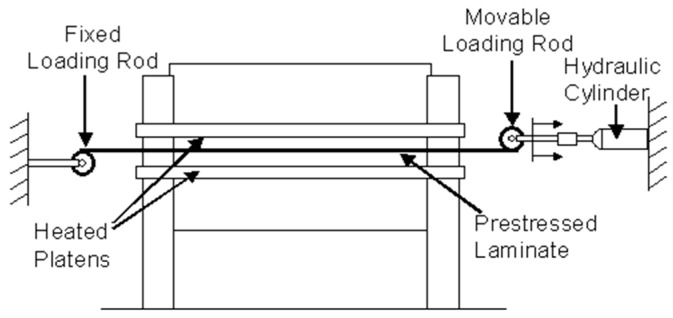
Schematic illustration of the hydraulic cylinder-based pre-stressing rig used by Tuttle *et al.* [38].

This is a variation on the dead-weight loading method illustrated in Figure 1 where Tuttle *et al.* [38] used a hydraulic device to pre-stress prepregs. The processing of the preform was carried out on a hot-press as illustrated in Figure 4. The hydraulic loading fixture was mounted on a horizontal frame, such that the pre-stressed plies remained parallel to the heated platens of the hot-press. One end of the preform was attached to a fixed loading-rod, and the opposite end was connected to the hydraulic ram through which the desired load was applied to the prepregs. They reported that in the initial experiments, slippage occurred between the ply and loading-rod surface at high fibre pre-stress levels; the slippage was eliminated by knurling the surface of the loading rods. The load applied to the fibres was monitored using a pressure gauge that was attached on the hydraulic ram line.

### 2.5. Horizontal Tensile Loading Machine

**Figure 5:**
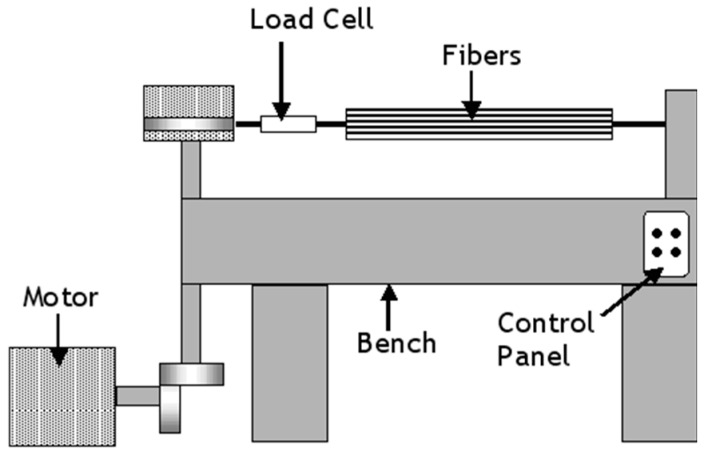
Schematic illustration of the horizontal tensometer-based pre-stressing rig used by Motahhari and Cameron [39].

Motahhari and Cameron [39] produced pre-stressed composites with the aid of a horizontal tensile testing machine as illustrated in Figure 5. In their design, the ends of the fibres were secured using clamps and the applied load was monitored via a load-cell. A U-shaped mold was used to manufacture the composites. A similar method was used by Lee [40] and Ali [41] to pre-stress prepreg. This pre-stressing technique was capable of measuring the magnitude of the applied pre-load during the curing process.

### 2.6. Fibre Pre-Stressing Rig

**Figure 6:**
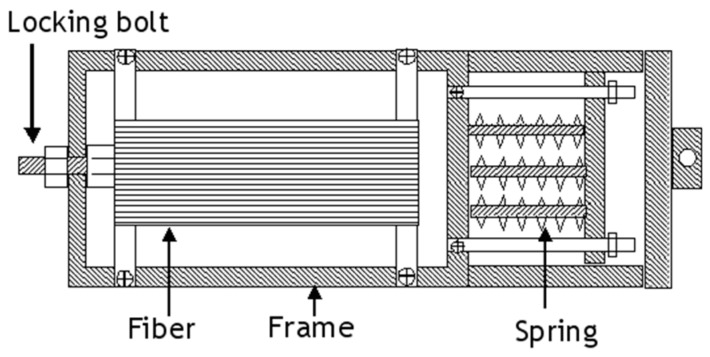
Schematic illustration of the fibre pre-stressing rig developed by Zhao and Cameron; this rig was used in combination with a hot-press [42].

The pre-stressing rig design developed by Zhao and Cameron [42] is illustrated in Figure 6 where the fibres were first wound onto a steel frame and then it was attached to a secondary frame. The pre-stress loading of the fibres on the frame was achieved via a tensile test machine. Once the required load was reached, locking-bolts were used to maintain the fibres in the stressed state. The pre-stressed preform was cured using a hot-press.

### 2.7. Biaxial Loading Frame

**Figure 7:**
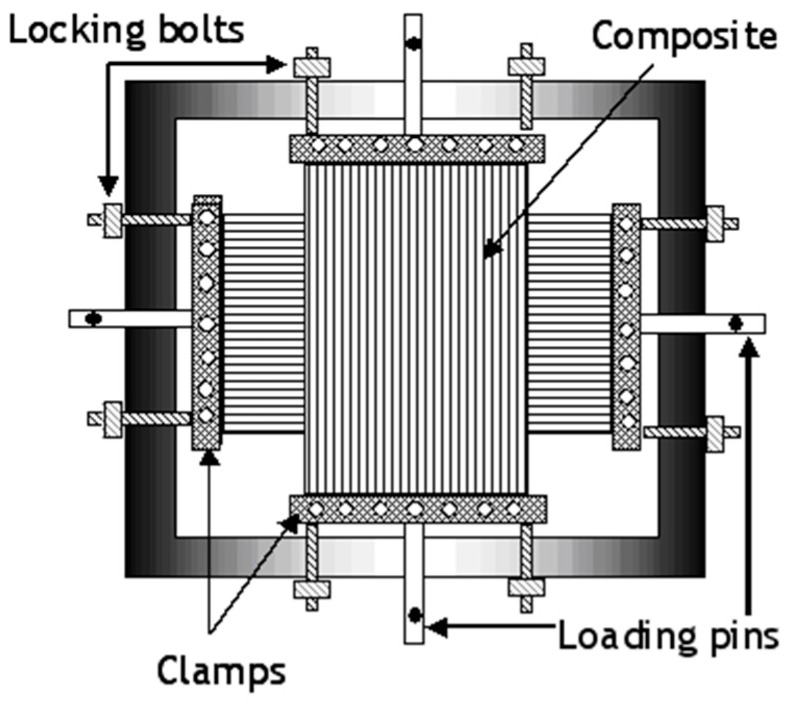
Schematic illustration of the biaxial loading frame-based prepreg pre-stressing method [43].

Jevons *et al.* [43] adapted the design originally proposed by Zhao and Cameron [42] and developed a method for pre-stressing cross-ply laminates that could be processed in an autoclave. Their design consisted of C-channel sections with four clamps linked to the frame by bolts as illustrated in Figure 7. The intended end-tab regions, at the ends of the laminated prepregs, were cured in a hot-press and end-tabbed. Several 10 mm diameter holes were drilled through the end-tabs and this assembly was bolted on to the loading frame. The required pre-stress was applied through the loading pins using a mechanical test machine; the locking-bolts between the clamps were then tightened when the desired level of pre-load was applied to the prepregs. The frame with the pre-stressed prepregs was vacuum-bagged and processed in an autoclave.

In conclusion, a number of ingenious devices have been used for manufacturing pre-stressed composites. The following sections report on the design and development of a fibre pre-stressing technique that permitted the preforms to be processed in an autoclave. The development of the residual strain in the composite during processing was monitored in-situ using embedded optical fibre sensors [44].

## 3. Materials and Methods

### 3.1. Pre-Stressing Rig Design

The primary design requirements for the pre-stressing rig were as follows: (i) a means to apply and monitor the magnitude of the pre-stress on the preform during and after processing; (ii) a facility to enable optical fibres to be integrated into the preform and for the entry/exit points to be protected; and (iii) processing the pre-stressed preforms in an autoclave using conventional vacuum bagging procedures.

**Figure 8:**
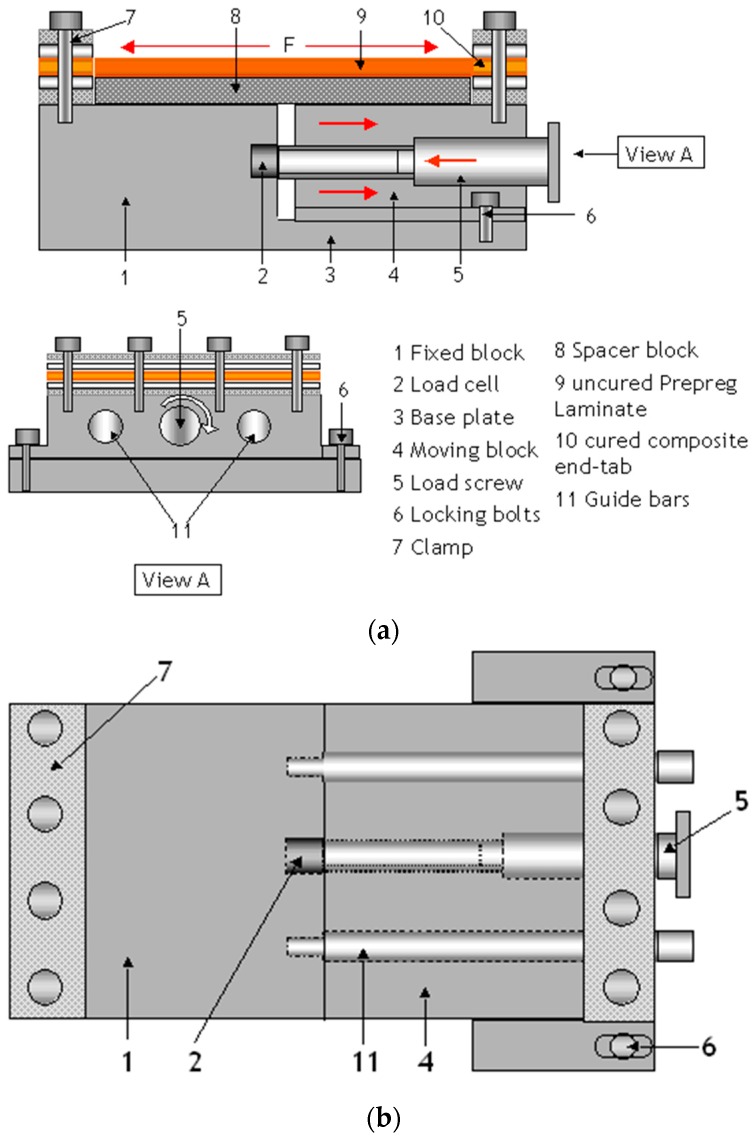
(**a**) Side-elevation of the flatbed rig for applying the desired pre-stress on laminated prepregs; (**b**) Top-elevation of the flat-bed pre-stressing rig.

Schematic illustrations of the flat-bed pre-stressing rig design are presented in Figure 8a,b. The basic mode of operation of the rig is the controlled separation of two sections of the flat-bed with the aid of load-screws. With reference to Figure 8a, two blocks serve as the flat-beds of which one is fixed to a base-plate and the other is able to slide on guide bars. The guide bars are attached to the fixed block as shown in Figure 8b. These guide bars also aid in the alignment of the clamps of the movable and fixed blocks. The applied load on the preform (when it is clamped in the rig) is monitored via a load-cell that is housed within a recess on the fixed block as illustrated in Figure 8b. The end of the load-screw is positioned in contact with the load-cell such that it is capable of transmitting the applied pre-stress on the laminated prepregs. The locking bolts illustrated in Figure 8a,b are used to lock the position of the movable block and this is achieved by clamping it to the base-plate after applying the required pre-stress to the end-tabbed prepregs.

The load-cell used in this study was Model No: 060-4771-01-08, RDP Electronics Ltd, (Wolverhampton, UK). The capacity of the load cell was 44.5 kN in compression and it was rated for operations up to 121 °C. The load-cell was calibrated by applying a static compressive load using a pre-calibrated Instron mechanical test machine, model 8501 (Instron, High Wycombe, UK). The calibration test was conducted at room temperature and the rate of loading was 0.125 kN/s. Static compression tests were conducted at 70, 100 and 120 °C and these tests were carried out using a mechanical test machine (Instron model 1195) equipped with an air-circulating oven.

### 3.2. Evaluation of the Pre-Stressing Rig

**Figure 9:**
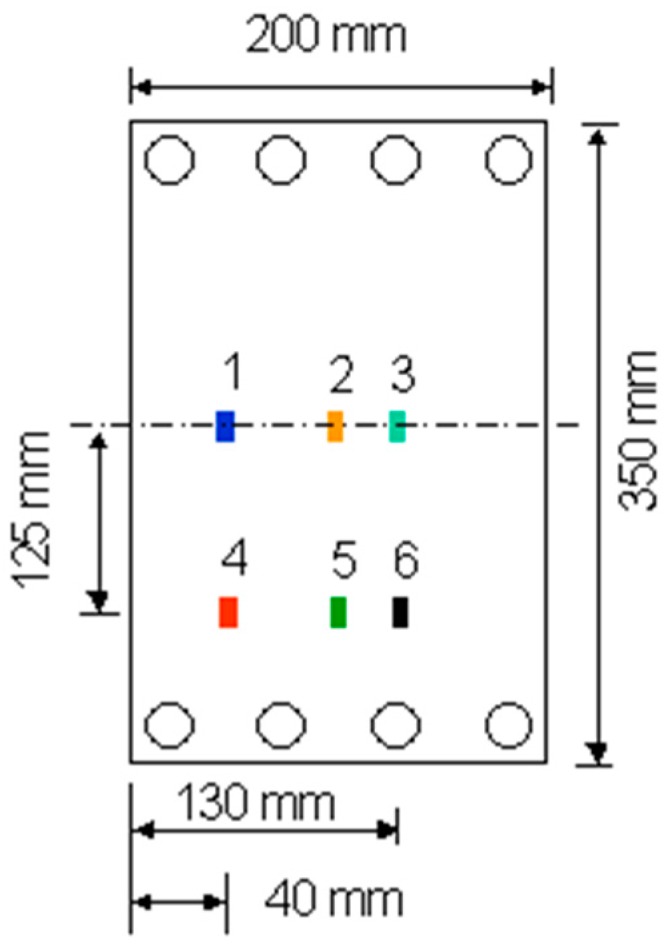
Schematic illustration of the location of the surface-mounted electrical resistance strain gauges that were used to monitor the strain on a steel plate when it was loaded on the flat-bed pre-stressing rig.

The pre-stressing rig was evaluated initially by clamping a steel plate and loading it in tension. The steel plate was instrumented with an array of surface-mounted electrical resistance strain gauges as shown in Figure 9. Mechanical loading was applied to the steel plate via the load-screw as described previously. The magnitude of the applied load and strain were monitored using the load-cell on the rig and the surface-mounted electrical resistance strain gauges respectively.

### 3.3. Fibre Optic Sensors

The optical fibre sensors used in this study were (i) extrinsic fibre Fabry-Perot interferometric (EFPI) strain sensors and (ii) fibre Bragg grating (FBG) strain and temperature sensors. Single-mode optical fibres designed for use at 800 nm and 1500 nm (SM 800 and SM 1500, Fibercore Ltd., Southampton, UK) were used to fabricate the sensors. The EFPI sensors with cavity lengths in the range 50 to 100 µm were fabricated in-house using SM 800 (5.6/125 µm) optical fibres and 128 µm precision bore fused silica capillaries. The details of the fabrication process and the mode of operation of the sensors have been published previously [44,45,46]. The FBGs were inscribed on SM 1550 (9/125 µm) optical fibres using the phase mask technique in conjunction with an excimer laser operating at 248 nm (Bragg Star).

#### 3.3.1. Interrogation of the EFPI Strain Sensor

A super-luminescent diode (SLD) operating at a center wavelength of 850 nm was used to illuminate the EFPI sensor and the resulting interference fringes were detected using a CCD spectrometer (HR 2000, Ocean Optics, Oxford, UK). The absolute cavity length was measured from the interference spectrum using the following equation:

(1) $$d=\frac{\lambda_{1}\lambda_{2}}{2.\Delta \lambda }$$

where λ_1_ and λ_2_ are the wavelengths corresponding to the maximum intensities of two adjacent bright fringes and Δλ is the free spectral range. The longitudinal strain on the sensor is expressed as the ratio of the change in cavity length to the gauge length of the sensor.

#### 3.3.2. FBG Strain and Temperature Sensor

The FBG sensors were monitored using a Bragg interrogation unit (Fiberpro Ltd., Dae-Jeon, Korea) equipped with an inbuilt wavelength sweep laser and a detector. The influence of strain (ε) and temperature (T) on a fibre Bragg grating can be expressed as [47]:

(2) $$\begin{array}{c} \frac{\Delta \lambda_{B}}{\lambda_{B}}=\left(\alpha_{fibre}+\eta \right)\Delta T+(1-p_{e})\varepsilon \\ \lambda_{B}\;(=2\Lambda n_{\mathrm{eff}}) \end{array}$$

where λ_B_ (=2Λn_eff_) is the Bragg resonance wavelength; α_fibre_ is the coefficient of thermal expansion of the fibre; n_eff_ is the effective index of the core and Λ is the grating period; *p*_e_ is the photo-elastic constant of the fibre; and *η* is the thermo-optic coefficient of the core. The response of the temperature sensing FBGs can be expressed as:

(3) $$\Delta T=\frac{\Delta \lambda_{B}}{\lambda_{B}(\alpha_{fibre}+\eta )}$$

The FBGs used in this work were produced using the same phase mask and laser irradiation conditions. The temperature sensor was produced by encapsulating a FBG inside a fused capillary in a strain-free condition whilst an unprotected FBG was used to monitor the strain and temperature. The axial strain on the unprotected FBG was calculated by decoupling the wavelength shifts obtained from the FBG temperature sensor.

### 3.4. Preparation of Pre-Stressed Composites

**Figure 10:**
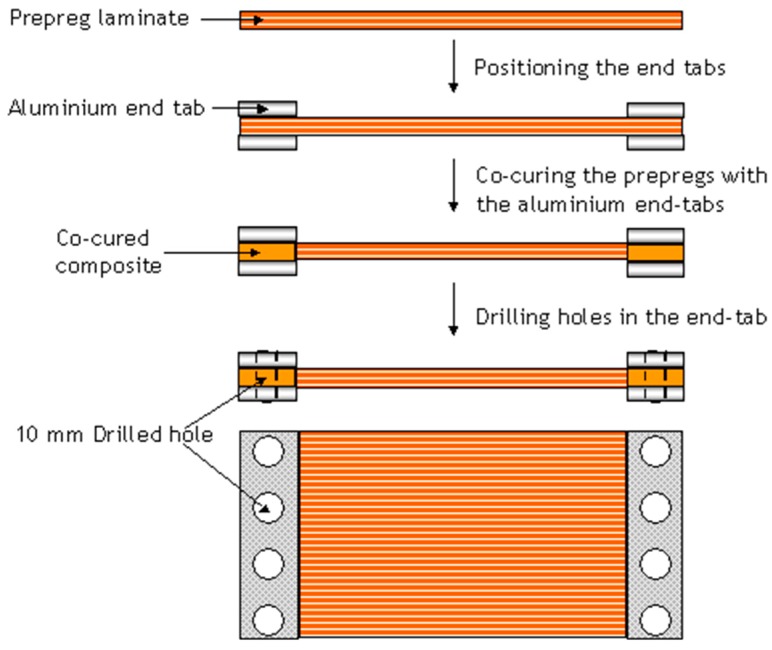
Schematic illustration depicting the sequence of operations that were carried out to co-cure the aluminium end-tabs on to the prepregs (within the end-tab region).

16-plys of unidirectional prepregs were laminated using conventional procedures. In order to apply a pre-stress to the laminated prepregs, it was necessary to attach end-tabs. This was carried out by co-curing aluminum end-tabs onto the designated areas in the preform. A schematic illustration of the sequence of operations associated with the preparation of the end-tabbed preform is shown in Figure 10.

**Figure 11:**
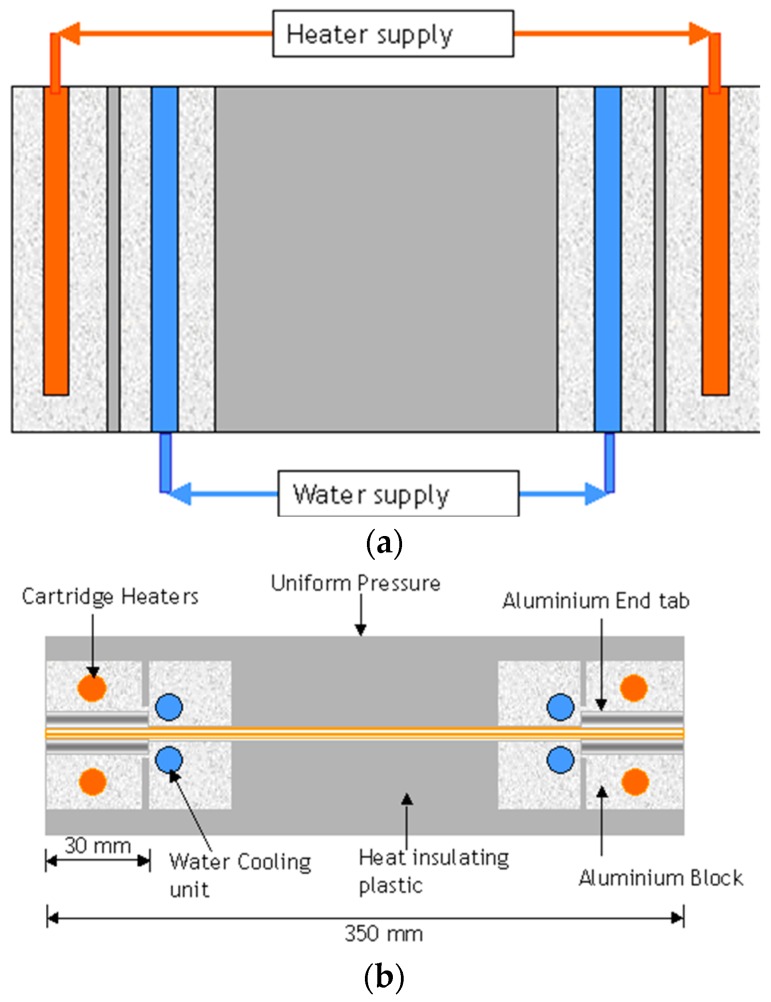
(**a**) Plan-view of the cartridge-based heating of the end-tabbed region and the water-based cooling of the preform in the vicinity of the heated region; (**b**) Side-view of the heating/cooling systems used in the production of the end-tabbed preform.

It was necessary to cool the section of the preform in the immediate vicinity of the end-tab during the co-curing process; this was done to ensure that the curing only occurred within the end-tabbed regions. Schematic illustrations of this jig to cool the preform outside the end-tabbed region are shown in Figure 11a,b. Water from a mains-tap was circulated through the rig and the output was directed to a sink.

Aluminium alloy (5251-H2, Metalfast Ltd., Swindon, UK) end-tabs were cut to size (30 (l) mm × 200 (w) mm × 1.5 (t) mm) and abraded on both sides. They were then degreased with acetone and dried. The end-tabs were aligned with the ends of the preform and cured in a hot-press at 120 °C for 1 h. After the end-tabs were co-cured on the ends of the preform, 10 mm diameter holes were drilled at regular intervals to enable it to be clamped to the pre-stressing rig. Subsequent to drilling, the holes were cleaned using a de-burring tool to avoid puncturing the vacuum bag. Prior to clamping the end-tabbed preform to the pre-stressing rig, the rig was degreased and a coating of a silicone release agent (Rocol, PR, RS Components Ltd., Bristol, UK) was applied. The prepregs that were previously co-cured with the aluminium end-tabs were placed between the serrated clamp plates and bolted to the pre-stressing rig using M12 bolts. A torque of 20 Nm was applied to each of the bolts using a torque wrench.

The desired mechanical pre-stress was applied to the end-tabbed prepregs by means of the load-screw and the applied load was monitored via the load-cell. Once the required pre-stress was applied, the locking bolts were tightened to 20 Nm; this clamped the moving block to the base plate. The prepreg assembly was then vacuum bagged using conventional procedures and cured in the autoclave as outlined in Section 3.5.

**Figure 12:**
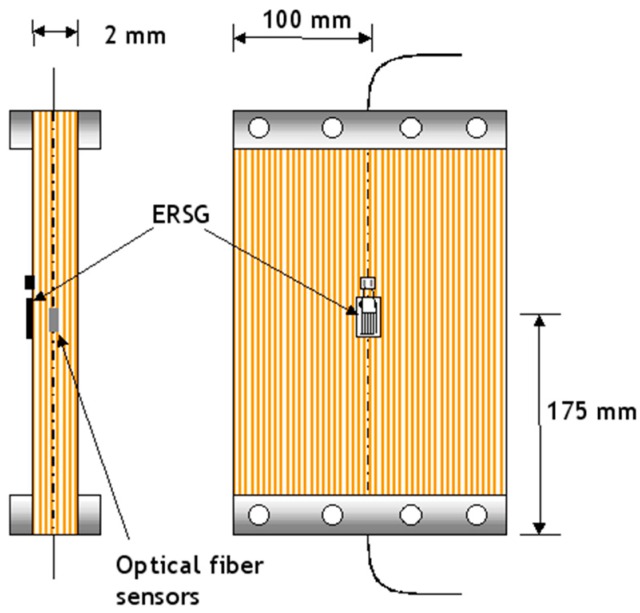
Side and plan-views of the relative locations of the surface-mounted electrical resistance strain gauges and the embedded optical fibre sensors.

During curing, the strain development in the prepregs/composite was monitored in-situ using the EFPI and FBG sensors. After curing and cooling to room temperature, the vacuum bag was removed. ERSG sensors were bonded on the surface of the composite in such a manner that that they were above the embedded optical fibres. A schematic illustration of the relative position of the ERSG is shown in Figure 12. The ERSG were bonded using cyanoacrylate adhesive (TML Ltd., Doncaster, UK). The previously induced fabrication strain in the cured composite was released by slackening the load-screw, locking bolts and the clamp bolts on the pre-stressing rig. The magnitude of the strain released upon unclamping the cured composite from the pre-stressing rig was recorded using the surface-mounted electrical and the embedded optical sensors.

### 3.5. Autoclave Processing of the Laminated Prepregs and the Pre-Stressing Rig Assembly

**Figure 13:**
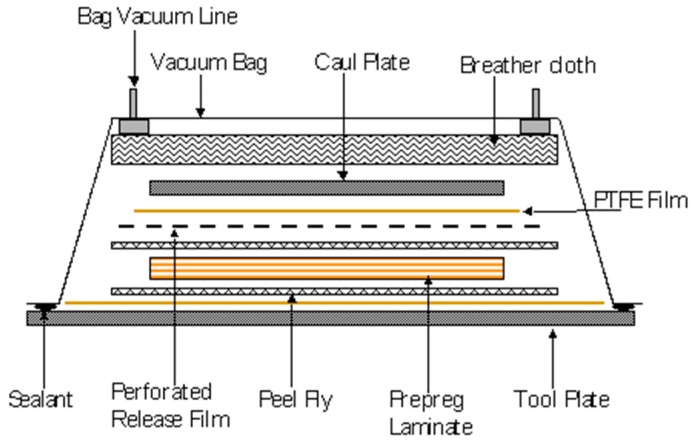
Schematic illustration of the key components of the materials contained within the vacuum bag in the autoclave (the pre-stressing rig is not shown).

A schematic illustration of the vacuum bagging assembly that was used for the production of the conventional composites is presented in Figure 13. A similar assembly was used for processing the pre-stressed prepregs in the pre-stressing rig.

The autoclave used in this study was custom-modified (Aeroform Ltd., Poole, UK) to include vacuum and pressure-rated input and output ports for the optical fibres to enable real-time strain and temperature monitoring. Prior to vacuum bagging the preform and the pre-stressing rig assembly, pre-shaped silicone blocks were packed around the rig to prevent puncturing of the vacuum bag when the vacuum was applied. The vacuum-bagged preform and the pre-stressing rig assembly were placed inside the autoclave and a vacuum (−0.084 MPa (−850 millibars)) was applied. The manufacturer’s recommended cure cycle for the E-glass/913 epoxy prepreg system was 1 h at 120 °C. The autoclave was programmed to heat up the vacuum bagged assembly at 2 K·min^−1^, with a pressure of 0.69 MPa (101 psi).

### 3.6. Characterising the Response of the Embedded Optical Fibre Sensors

A 16-ply UD E-glass/epoxy composite with embedded optical fibre sensors was used to assess the response of the embedded sensors in the autoclave to temperature, vacuum, pressure and the cure cycle. These tests were conducted using a laminate of dimensions 290 mm × 200 mm × 2 mm. The EFPI and FBG sensors were embedded between the 8th and 9th ply. After the laminated prepreg was processed, reference (unbonded) EFPI and FBG sensors were also located on the top-surface of the composite. The movement of the sensors was restricted by attaching the optical fibres (away from the sensing regions) to the surface of the composite using a high-temperature polyimide adhesive tape. A K-type thermocouple was also surface-mounted on the laminate/composite. The prepregs with the embedded optical fibre sensors were processed as described previously.

### 3.7. In-Situ Residual Strain Monitoring in Composites

The process-induced residual strain in unidirectional [0]_16_ E-glass epoxy composites, with and without pre-stress, was monitored *in-situ* using embedded EFPI and FBG sensors.

### 3.8. Coding of the Composites

In order to identify the composites with different levels of pre-stress, the following coding system was used:

(i) URX where U = unidirectional, R = reference (without pre-stress), X = panel number.

For example, UR6 represents unidirectional reference panel number 6.

(ii) UPTX where P = pre-stressed and T= pre-stressing rig, thermal expansion-induced pre-stress.
(iii) UPX_FkN where P = pre-stressed, FkN = pre-load in kN.

For example, UP6_14kN represents unidirectional pre-stressed panel number 6 with a 14 kN pre-load.

## 4. Results and Discussion

### 4.1. Calibration of the Load-Cell

**Figure 14:**
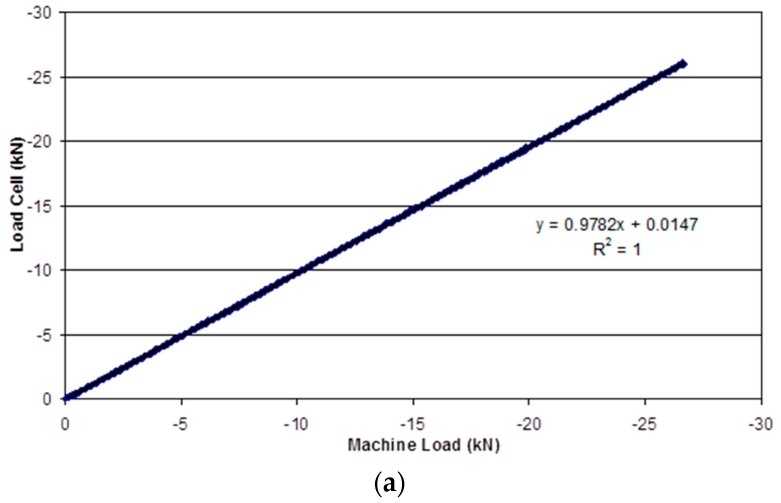

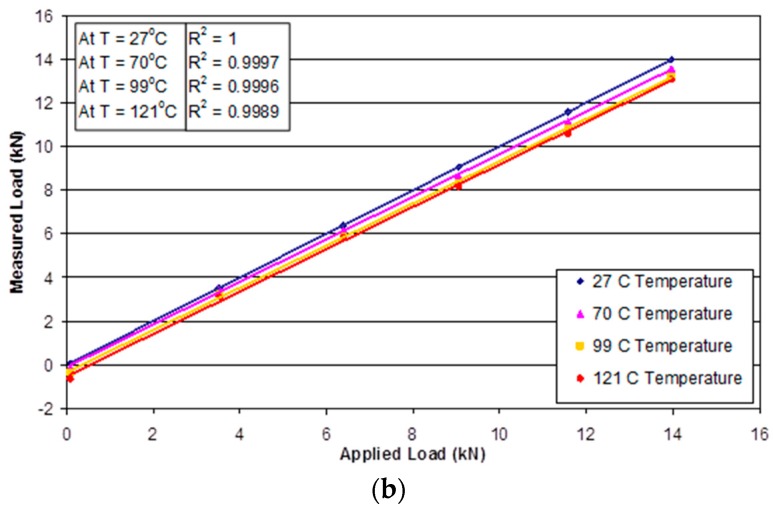
(**a**) Comparison of the load recorded by the load-cell (used on the pre-stressing rig) and the output from the pre-calibrated mechanical test machine; (**b**) Comparison of the load measured by the load-cell and the applied load via the mechanical test machine at specified temperatures.

With reference to Figure 14a, it is seen that the load recorded by the load-cell on the pre-stressing rig is in good agreement with that measured from the pre-calibrated mechanical test machine. Figure 14b shows that the response from the load-cell is linear during the static tests conducted at 27, 70, 99 and 121 °C.

### 4.2. Evaluation of the Pre-Stressing Rig

**Figure 15:**
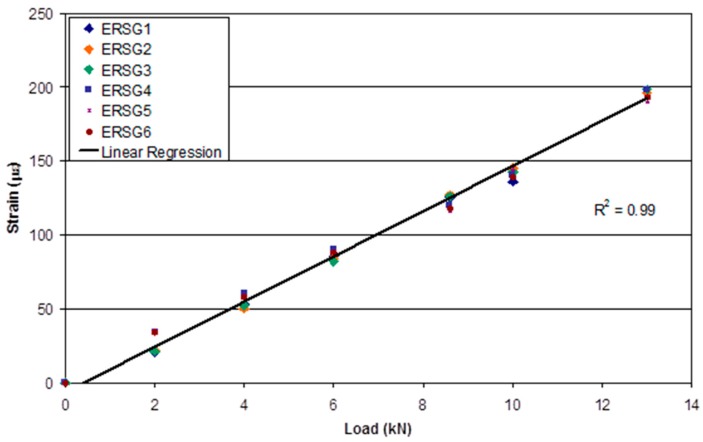
Strain measured on the steel plate via the surface-bonded ERSGs as a function of the applied load measured by the load-cell of the pre-stressing rig.

The loading efficiency of the pre-stressing rig was evaluated by clamping a steel plate and applying the required static tensile load. The resultant strain in the steel plate was measured using surface-bonded electrical resistance strain gauges (ESRGs). With reference to Figure 15, it can be seen that the strains measured at different positions on the steel plate, for an applied load, are in good agreement. This demonstrates that the clamping and loading mechanisms on the pre-stressing rig were capable of applying a uniform load to the steel plate.

### 4.3. Effect of the Autoclave Processing Parameters on the Sensors

**Figure 16:**
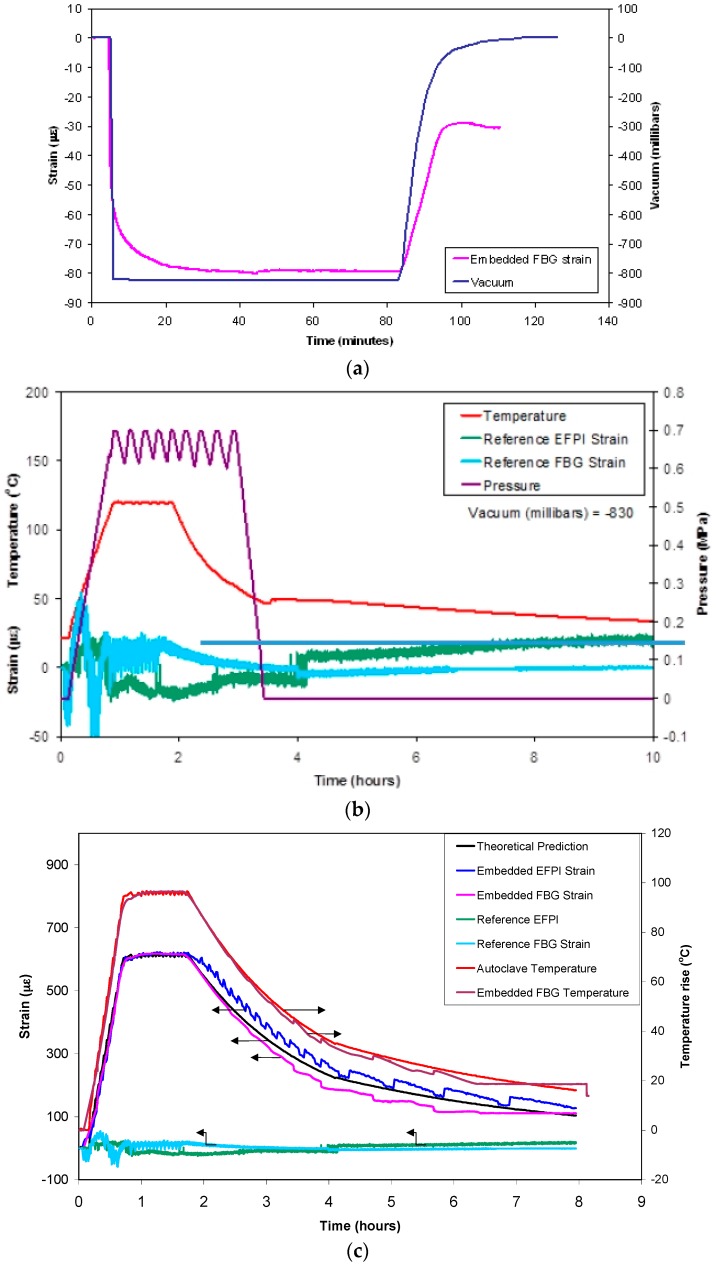
(**a**) The effect of the applied autoclave vacuum, via the vacuum bag, on an embedded FBG strain sensor; (**b**) Typical outputs from the surface-located reference EFPI and FBG sensors and the cure cycle parameters in the autoclave. The step in the FBG optical fibre sensor traces after approximately 4-h coincides with the time when the door of the autoclave was opened and the vacuum bag was detached.; (**c**) A comparison of the outputs from the embedded and surface-located EFPI and FBG sensors, including the data from the thermocouple, as a function of the cure schedule for a 16-ply unidirectional E-glass composite. The arrows indicate the relevant y-axis for each dataset.

The effect of the applied vacuum (826 mbar (−0.084 MPa)), via the vacuum bag, on an FBG that was embedded in a unidirectional laminate is shown in Figure 16a. A compressive strain of 80 µε was recorded for this applied vacuum. The interesting point to note is that the strain recorded by the FBG, when the vacuum bag was returned to atmospheric conditions, was approximately −35 µε.

The implication of the data presented in Figure 16a is that due care and attention needs to be paid to maintaining the relative orientation of the embedded optical fibre sensor in specified substrates (resin, prepreg, fabric, concrete, *etc.*). In the current paper, the alignment of the FBG and EFPI was controlled and assured because sections of the optical fibres, within the end-tabs, were pre-aligned and bonded to the matrix. The act of tensioning the end-tabbed preform, to apply the required pre-tension, ensured the alignment and the retention of the orientation of the optical fibre sensors during processing. 

The issues associated with the retention of the relative orientation of embedded sensors was highlighted previously [48]. The feasibility of measuring the residual strain using optical EFPI sensors was first demonstrated by Liu *et al.* [48] using a cross-ply carbon/epoxy composite. They embedded two temperature-compensated EFPI strain sensors between plies numbers 2 and 3 (sensor 1), and 8 and 9 (sensor 2) in a cross-ply (0, 90_2_, 0_2_, 90, 0, 90)_S_ carbon/epoxy laminate. They measured the cavity length of EFPI sensors before and after processing and reported the residual strain to be 90 µm and 550 µm in sensors 1 and 2 respectively. The authors proposed that the observed discrepancy may have been due to the relative orientations of the two sensors.

The effect of autoclave processing parameters on the output of the FBG and EFPI sensors (placed on the surface of the composite and embedded) was investigated using a pre-cured composite panel of dimensions 290 mm × 200 mm × 2 mm. The composite with the sensors was vacuum-bagged and subjected to a typical autoclave cure schedule. Figure 16b shows the observed relationship between the cure cycle parameters on the output from the reference EFPI and FBG sensors (located on the surface of the cured composite panel but within the vacuum bag). The FBG sensor was influenced by temperature, pressure and the force exerted by the vacuum bag. The initial fluctuations in the FBG signal, reaching a maximum compressive strain of approximately 40 µε, is likely to have been caused by the lateral compression caused by the application of the vacuum in the vacuum bag and the applied external pressure. At around 50 °C, the strain recorded by the FBG decreases from approximately +50 µε to a minimum of −50 µε. However, the signal is noisy since the mode of operation of the autoclave pressure is cyclic once the target value has been reached. It is difficult to account for the change in magnitude of the FBG signal from +50 to −50 µm. However, it is possible that some form of relaxation occurring in the lateral forces experienced by the fibre along its length. A possible reason for this observation is the glass transition temperature of the acrylate coating on the optical fibre being approached and/or being exceeded. In the current experiment, the acrylate coating was retained on the optical fibre apart from the sensing regions in the EFPI and FBG sensors. The compressive strain is reversed at approximately 80 °C and the FBG signal is seen to oscillate with the applied autoclave pressure to around +20 and −20 µε. After 2-h of processing, the heater controller was turned off and the strain recorded by the FBG is seen to decay in tandem with the temperature.

The EFPI sensor is relatively insensitive to temperature because the coefficients of thermal expansion for the optical fibre and the capillary are similar [45,46]. However, the action of the vacuum bag will impress the EFPI sensor on to the surface of the pre-cured composite as the vacuum and pressure is applied. This may account for the initial rise in the measured strain (~15 µε) after approximately 15 min and it could be attributed to the bending induced by the vacuum bag acting on the ends of the capillary and the optical fibre. A decrease is observed in the recorded strain from approximately 15 µε to 25 µε at 85 °C. This coincides with the autoclave pressure reaching its set value. Since this small but detectable decrease in the stain was observed for the FBG and EFPI sensors, it is speculated that this may be attributed to the glass transition temperature of the acrylate coatings being exceeded on the two types of optical fibres. After the autoclave door was opened, a small increase in the strain was observed from the reference EFPI sensor. This may be attributed to the relaxation of the materials within the vacuum bagging materials. However, this ceases after approximately seven hours as the autoclave was permitted to cool naturally.

The thermal expansion of a pre-cured unidirectional composite was measured using the embedded optic fibre sensors. The cured composite with the surface-located and embedded sensors were subjected to a typical autoclave cure cycle. The composite was heated from ambient to 120 °C at 2 K·min^−1^ with a dwell of one hour. The pressure was ramped from atmospheric to 0.69 MPa at 0.015 MPa per minute and held at this pressure for one hour.

The thermal expansion of a unidirectional composite (α_c_), in the fibre direction is given by:

(4) $$\alpha_{c}=\frac{\alpha_{m}\;\nu_{m}\;E_{m}\;+\;\alpha_{f}\;\nu_{f\;}E_{f}}{E_{m}\;\nu_{m}\;+\;E_{f}\;\nu_{f}}$$

where α, ν and E are the thermal expansion coefficient, volume fraction and modulus respectively. The subscript m and f denote the matrix and fibre respectively.

Figure 16c shows the output from the embedded, surface-located EFPI and FBG sensors and the thermocouple data as a function of the processing time and the autoclave temperature. In this instance, the strains recorded by the embedded EFPI and FBG sensors show a similar trend and magnitude during the heating and isothermal periods. During the cooling phase, a small divergence is seen between the EFPI and the FBG sensors.

Excellent correlation is seen in Figure 16c between the embedded FBG temperature sensor and the output from the thermocouple. The outputs from the reference EFPI and FBG sensors that were located on the surface of the composite were discussed previously. A good correlation is observed between the outputs from the embedded EFPI and FBG sensors and that predicted using Equation (4). A summary of the relevant properties that were used for computing the thermal expansion of the unidirectional 16-ply composite is presented in Table 1. The measured and the predicted values for the thermal expansions are presented in Table 2.

### 4.4. Residual Strain Monitoring in Composites without Pre-Stress

**Figure 17:**
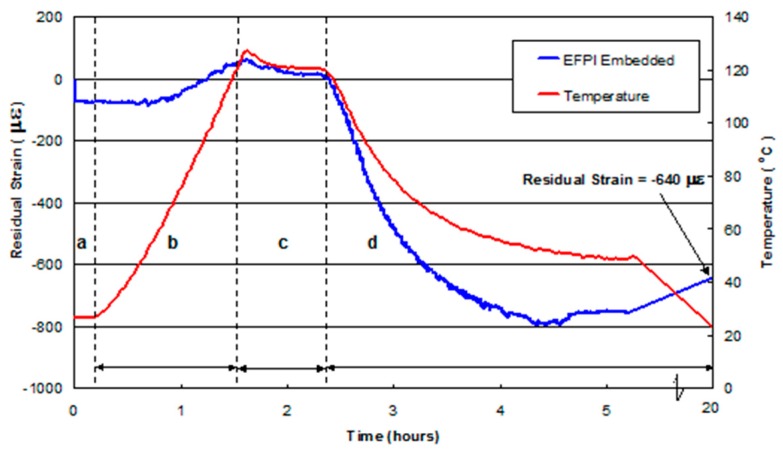
Strain measured using an embedded EFPI sensor in a reference [0]_16_ laminate (manufactured without any pre-stress) during autoclave processing. The coded regions (**a**–**d**) represent the follows: (**a**) before curing; (**b**) heating cycle; (**c**) dwell period; and (**d**) cooling cycle.

Figure 17 shows the output from an embedded EFPI sensor in a 16-ply UD E-glass/epoxy reference composite (manufactured without any pre-stress) during autoclave processing. A summary of the residual strains measured at specified stages of the cure cycle is presented in Table 3. The general trends observed in Figure 17 can be grouped into the following regions: (a) after embedding the EFPI sensor but before curing the laminated prepregs; (b) during the temperature ramp-up period; (c) during the isothermal dwell; and (d) cooling phase.

Region a: Subsequent to embedding the EFPI sensor, a compressive strain of approximately 50 µε was recorded. The origin of this compressive strain may be attributed to the manual procedures that were used to laminate and consolidate the prepregs with the aid of a roller. This action of consolidating the plies, layer-by-layer, is likely to have resulted in the EFPI with a capillary diameter of 300 µm, being impressed into the prepregs thus resulting in the observed compressive strain.

Region b: During the heating cycle, no significant changes were observed in the output of the EFPI sensor until approximately 60 °C when a positive strain was recorded. This increase may be attributed to a combination of the relaxation of the previously induced compressive strain due to the lamination process and thermal expansion of the various materials. During the heating phase, the viscosity of the resin initially decreases. Hence, it is reasonable to assume that any constraint or bending introduced on the optical fibre and sensor during lamination will relax from its constrained position. The viscosity/temperature profile for the Fiberdux 913 resin system reported by the manufacturer indicated that the minimum viscosity is reached between 120–130 °C. After this period, the viscosity of the resin increases as the cross-linking reactions become prominent. This cross-linking process is accompanied by shrinkage of the resin. However, as the temperature in the autoclave is increasing in region-b, and therefore, the constitutive materials in the composite and the pre-stressing rig undergo thermal expansion.

Region c: The observed excursion of approximately 7 °C at the end of the temperature ramp is possibly due to two factors. Firstly, it could be a consequence of the exothermic ring-opening cross-linking reaction between the epoxy resin and amine hardener. Secondly, it could be due to the temperature-controller in the autoclave overshooting the set isothermal value. On inspecting region-c in Figure 17, it is apparent that preforms were above the set isothermal temperature for approximately 20 min from the end of the thermal ramp. This exothermic excursion was observed for all the prepregs processed in this study.

Region d: Here the heating was turned off and the composite was permitted to cool naturally.

With reference to Table 3, the average residual strain measured from three UD reference composites, at ambient temperature, was –604 µε.

### 4.5. Residual Strain Development in a UD Pre-Stressed Composite

This section reports on the use of embedded EFPI sensors in unidirectional [0]_16_ E-glass epoxy composites where the preform was pre-stressed. The EFPI sensors were used to monitor the strain during and after processing. A limited number of experiments were also undertaken using embedded FBG sensors. In addition, electrical resistance strain gauges were surface-bonded on the composite after processing but before they were unclamped from the pre-stressing rig. The magnitude of the strain released, when the composite was unclamped from the pre-stressing rig, was recorded using the embedded optical fibre sensors and the surface-bonded ERSGs.

**Figure 18:**
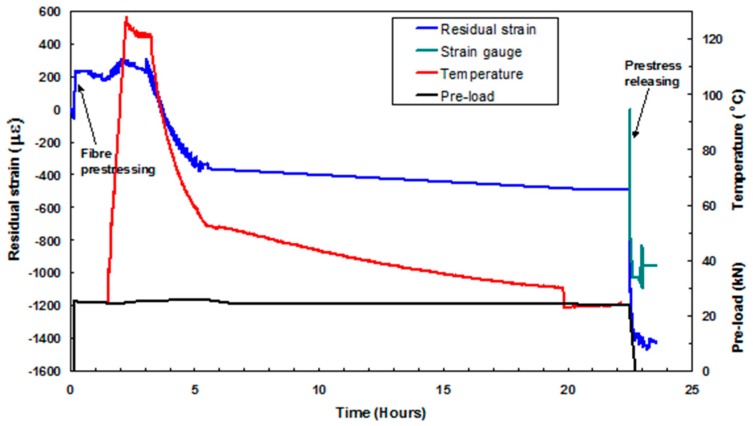
Strain development throughout the processing of a 16-ply UD prepreg system that was subjected to a pre-stress of 150 MPa.

Figure 18 shows the outputs from the various sensors when the composite, with a previously applied pre-stress of 150 MPa, was unclamped from the pre-stressing rig. It can be seen that the applied pre-load was maintained throughout the curing process to within 1 kN. During the heating cycle, the load-cell did not record the pre-stress induced in the preform due to thermal expansion of the rig. This is because in the current set-up, there is no frame of reference for the load-cell to record the thermally-induced pre-stress experienced by the preform. During the heating phase, the pre-stressing rig assembly expands and this will induce a pre-stress on the preform. However, the load applied to the preform due to thermal expansion of the rig will not be recorded by the load-cell.

On inspecting the output from the EPPI sensor in Figure 18, it is seen that a compressive strain of approximately 60 µε is induced in the preform prior to the application of the pre-load. As expected, the application of a pre-stress 150 MPa resulted in an increase in the strain recorded by the EFPI sensor. The residual strain and the magnitude of the strain that was released when the composite was unloaded from the pre-stressing rig, for four pre-stress levels, are summarised in Table 4 and Table 5 respectively.

With reference to Table 5, the EFPI strain data from the repeat experiments, at pre-loads corresponding to 14 kN, were in the range −537 to −637. A reasonable agreement is observed between the datasets for the EFPIs and the ERSGs when they were embedded and surface-mounted respectively, at the centre of the composite. The ERSGs, when located 40 mm from the edge of the composite, recorded a higher value when compared to those located at the centre; this is presumably due to edge effects. Reasons for the variation in the FBGs were discussed previously.

**Figure 19:**
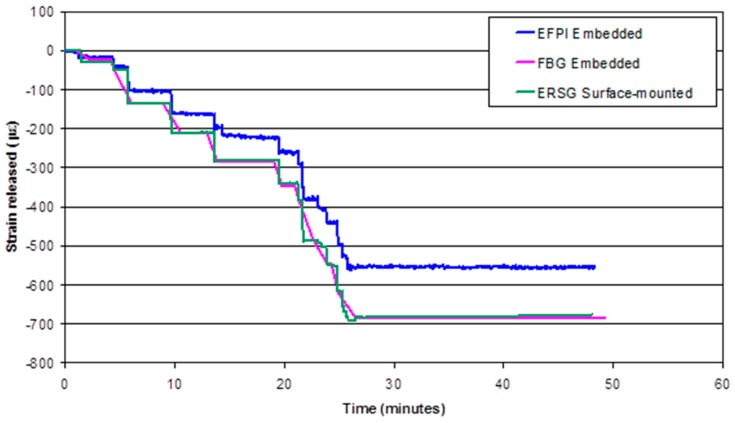
Strain data from the EFPI, FBG and ERSG sensors when a composite (Panel code UP9_14kN), with a previously applied pre-stress of 108 MPa, was unloaded from the pre-stressing rig in a stepwise manner.

Figure 19 shows the strains recorded by the EFPI, FBG and ERSG sensors when a composite (Panel code UP9_14kN), with a previously applied pre-stress of 108 MPa, was unloaded from the pre-stressing rig in a stepwise manner. The data recorded by the FBG sensor did not follow the stepwise pre-load release as observed with the EFPI and ERSG sensors. This is because the rate of data acquisition for FBG sensors was two measurements per minute, whereas that for the EFPI and ERSG sensors was 1 measurement per second. Initially, the strains measured from the EFPI, FBG and ERSG sensors are in agreement. However, as the composite was unloaded further, the EFPI sensor records a slightly lower compressive strain when compared to the FBG and ERSG sensors. On the other hand, the FBG and ERSG sensors are in good agreement.

Table 6 shows the average strain-release measured via the EFPI, FBG and ERSG sensors when the preform that was previously subjected to a pre-stress 108 MPa, was cured and then unloaded from the pre-stressing rig. The average strain-release measured with the FBG sensor is slightly higher than that obtained from the EFPI sensor. However, by considering the standard deviations and the number of samples that were evaluated, it can be concluded that the strain recorded from the optical fibre (EFPI and FBG) and ERSG sensors show a reasonable agreement. After releasing the pre-stress, the final residual strain in the composite, ε_final_, can be expressed as:

(5) $$\varepsilon_{final}=\;\varepsilon_{r}-\;\varepsilon_{p}$$

where *ε_r_* is the residual strain measured before releasing the composite from the pre-stressing rig (this represents cure and thermally-induced residual stresses) and *ε_p_* is the strain measured when the pre-load is released.

**Figure 20:**
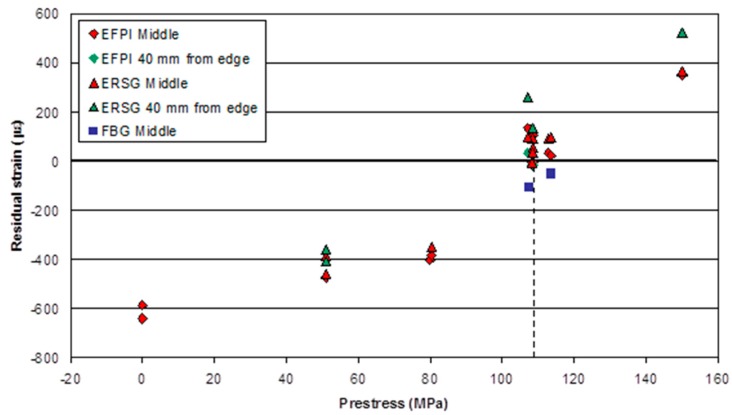
The measured residual strains in the composites produced in this study as a function of the pre-stress levels that were applied to the prepregs prior to curing.

Figure 20 shows the final residual strain measured for all the pre-stressed composites manufactured in this study. The compressive residual strain in the composites reduces as the previously applied pre-stress on the preforms increases. However, the application of a pre-stress to the preform of approximately 108 MPa results in the production of composites with a near-zero residual strain. Above an applied pre-stress of 108 MPa, the final residual strain is tensile. This demonstrates that the pre-stressing method reported here can be used to control the final residual strain in composites.

In Figure 20, it is apparent that the strain gradient across the panel (middle and 40 mm from edge) increases with an increase in pre-stress. This variation in strain reaches a maximum of 14% at a 150 MPa pre-stress. It is proposed that this difference in the measured strain-release may be due to an edge effect caused by the variation in Poisson’s contraction at the unconstrained edge.

## 5. General Discussion

This study had demonstrated conclusively that the application of a pre-stress to the preform, and maintain this stress during autoclave-based processing, is a viable method for counteracting the fabrication-induced residual strain in unidirectional composites. In addition to the parameters discussed in the introduction that can give rise to residual fabrication strain in composites, attention also needs to be paid to non-uniform temperature and cure gradients during the processing of preforms [49,50]. With reference to strain metrology using optical fibres, due consideration needs to be given to the nature of the interface between the surface of the optical fibre/sensor and the matrix [1,51,52]. Appropriate packaging and sensor protection systems are also important if the longevity of the sensor system is to be ensured [53,54].

Although the optical fibre sensors were effective in quantifying the magnitude of the residual strains in pre-stressed composites, for the first time, the reasons for the variations in the strain data need to be appreciated. Optical fibres can be embedded with significant ease in prepregs, however, due attention needs to be paid to the orientation of the sensor in relation to the reinforcing fibres [1]. Any bending of the optical fibres, in the vertical or horizontal planes to the reinforcing fibres, will give rise to induced strain in the sensor. In general, in-plane movement of the sensor is possible when there is significant movement of the resin during processing. This in-plane movement can be induced by excessive resin-flow during processing or if excessive pressure is applied. This movement of the sensor can be restricted by securing the input and output ends of the optical fibres under a pre-defined tension.

The thermal expansion of the substrate that the optical fibres are attached to could also have an influence on the measured strain via the sensors. Out-of-plane movement of the optical fibre sensor has been observed for unidirectional composites [1]. However, a detailed study needs to be undertaken to assess the effect of processing conditions on the relative movement of the optical fibre sensors; due attention also needs to be paid to the effect of the optical fibres on the orientation of the reinforcing fibres. The issue here is that the localised strain measured by the optical fibre sensor may not give an accurate description of the strain. Instrumental drift can be a problem especially if the laboratory or environment housing the interrogation equipment experiences a significant change in temperature.

The effect of the autoclave processing parameters (pressure, vacuum) needs to be quantified for a specific prepreg system. The FBG is more sensitive to lateral loading than the EFPI sensor. This current study has shown that more reliable data can be obtained from the FBG sensor when reflection spectra are recorded as opposed to analyses where only the peak-reflection is monitored. The latter can give rise to erroneous conclusions especially where the sensing region experiences off-axis loading.

## 6. Conclusions

This study has demonstrated the feasibility of manufacturing unidirectional composites where the magnitude of the residual stress can be managed by applying a pre-stress to the preform prior to processing. This was achieved using a custom-designed pre-stressing rig that was based on a two-part flat-bed design. This rig permitted the pre-stressed prepregs to be processed in an autoclave using conventional procedures. The feasibility of using conventional EFPI and FBG sensors for monitoring the development of residual strain was also demonstrated. The evaluation of the pre-stressing technique was carried out in four stages. Firstly, the load-cell was calibrated at specified applied loads and temperatures. Subsequent to this, the clamping/loading efficiency was demonstrated by loading a steel plate in the rig. The resultant strain was logged at specified positions using surface-bonded ERSGs. Secondly, an end-tab rig was designed to enable aluminium end-tabs to be co-cured to the ends of the prepregs but without curing the regions away from the end-tabs; this was achieved by using a water-cooling system. Thirdly, the effect of the autoclave processing parameters on the outputs from the sensors used in this investigation was studied. Finally, composites were manufactured with predefined levels of pre-load and the effect of pre-stress on the residual stress in composites was determined.

The residual strain in unidirectional E-glass glass fibre/epoxy composite panels was measured using embedded EFPI and FBG sensors. This study has shown conclusively that the magnitude of the residual stress in a composite can be managed by the application of pre-stress during processing. For example, it was demonstrated that applying a pre-stress of 108 MPa to a unidirectional E-glass epoxy preform, resulted in a composite panel with a negligible residual strain.

## Figures and Tables

**Table 1:** A summary of relevant properties that were used for computing the thermal expansion of the unidirectional 16-ply E-glass/epoxy resin composite.

Properties	E-Glass	Epoxy Resin
Young‘s Modulus (Mpa)	72,500	4000
Poisson‘s ratio	0.22	0.4
Thermal expansion coefficient (°C^−1^)	5.00 × 10^−6^	60.0 × 10^−6^
Volume fraction	0.6	0.4

**Table 2:** A summary of the measured and predicted thermal expansions for the unidirectional 16-ply E-glass/epoxy resin composite.

Method	Thermal expansion (×10^−6^ K^−1^)
Theoretical prediction (Equation (4))	6.38
Experimental measurement (EFPI and FBG)	6.45

**Table 3:** A summary of the strain data measured by the embedded EFPI sensors at different stages of processing for three reference composite panels where no pre-stress was applied.

Reference Composite Panels	Strain Before Embedding (µε)	After Embedding (µε)	At 120 °C (µε)	Final Residual Strain at Room Temperature (µε)
UR1	0	−71	40	−587
UR8	0	−63	42	−640
UR9	0	−101	24	−586

**Table 4:** EFPI and FBG-based strain data from unidirectional composites that were pre-stressed via thermal expansion of the rig, and applied pre-load values on the preforms corresponding to 7, 14 and 24 kN. The strain data were acquired at room temperature.

Composite Panel Code	Residual Strain (µε)		
	Sensor Type and Position on the Panel		
	EFPI (Middle)	FBG (Middle)	EFPI (40 mm from the Edge)
UPT8	−566	-	-
UPT11	−581	-	-
UP1_7kN	−586	-	-
UP2_7kN	−574	-	-
UP2_14kN	−564	-	−643
UP3_14kN	−503	-	−653
UP4_14kN	−573	-	-
UP5_14kN	−553	-	-
UP6_14kN	−520	-	-
UP7_14kN	−605	-	-
UP9_14kN	−529	−742	-
UP12_14kN	-	−714	-
UP1_24 kN	−592	-	-

**Table 5:** Strain recorded from the EFPI, FBG and surface-mounted ERSG sensors when the pre-stress was released by unclamping the composite from the pre-stressing rig. The strain data were acquired at room temperature.

Composite Panel Code	Pre-Stress Released (MPa)	Strain Released Upon Unloading the Composite from the Pre-Stressing Rig (µε)				
		Sensor Type and Position on the Panel				
		EFPI (Middle)	FBG (Middle)	EFPI (40 mm from the Edge)	ERSG (Middle)	ERSG (40 mm from the Edge)
UPT8	51.21 *	-	-	-	−109	−160
UPT11	51.21 *	−108	-	-	−194	−221
UP1_7kN	79.95	−184	-	-	-	-
UP2_7kN	80.36	−190	-	-	−223	-
UP2_14kN	108.50	-	-	−630	−656	−696
UP3_14kN	107.22	−637	-	−685	−602	−763
UP4_14kN	108.62	−579	-	-	−628	-
UP5_14kN	108.41	−537	-	-	−548	-
UP6_14kN	112.89	−552	-	-	−613	-
UP7_14kN	108.45	−600	-	-	−639	-
UP9_14kN	113.57	−551	−684	-	−677	-
UP12_14kN	107.61	-	−608	-	−600	-
UP1_24 kN	150.07	−944	-	-	−958	−1113

*: The thermal expansion-induced pre-stress calculated using classical mechanics.

**Table 6:** The average strain released when the composites were un-loaded from the pre-stressing ring. The preforms were previously subjected to a pre-stress of 108 MPa.

Sensor Type	Number of Samples	Average Strain Released Upon Unloading (µε)	Standard Deviation (µε)
EFPI	6	−592.8	56
FBG	2	−646.0	54
ERSG	7	−616.2	35

## Abbreviations

The following abbreviations are used in this manuscript:

AFRCs	Advanced Fibre Reinforced Composites
CCD	Charged coupling device
EFPI	Extrinsic Fibre Fabry-Perot interferometer
ERSG	Electrical resistance strain gauges
FBG	Fibre Bragg grating
ILSS	Interlaminar shear strength
SLD	Super-luminescence diode
SMA	Shape memory alloy
